# DeepVID v2: self-supervised denoising with decoupled spatiotemporal enhancement for low-photon voltage imaging

**DOI:** 10.1117/1.NPh.11.4.045007

**Published:** 2024-10-29

**Authors:** Chang Liu, Jiayu Lu, Yicun Wu, Xin Ye, Allison M. Ahrens, Jelena Platisa, Vincent A. Pieribone, Jerry L. Chen, Lei Tian

**Affiliations:** a Boston University, Department of Biomedical Engineering, Boston, Massachusetts, United States; b Boston University, Department of Electrical and Computer Engineering, Boston, Massachusetts, United States; c Boston University, Department of Computer Science, Boston, Massachusetts, United States; d Boston University, Neurophotonics Center, Boston, Massachusetts, United States; e Boston University, Department of Biology, Boston, Massachusetts, United States; f Yale University, Department of Cellular and Molecular Physiology, New Haven, Connecticut, United States; g The John B. Pierce Laboratory, New Haven, Connecticut, United States; h Yale University, Department of Neuroscience, New Haven, Connecticut, United States

**Keywords:** deep learning, self-supervised denoising, voltage imaging, low photon, microscopy

## Abstract

**Significance:**

Voltage imaging is a powerful tool for studying the dynamics of neuronal activities in the brain. However, voltage imaging data are fundamentally corrupted by severe Poisson noise in the low-photon regime, which hinders the accurate extraction of neuronal activities. Self-supervised deep learning denoising methods have shown great potential in addressing the challenges in low-photon voltage imaging without the need for ground-truth but usually suffer from the trade-off between spatial and temporal performances.

**Aim:**

We present DeepVID v2, a self-supervised denoising framework with decoupled spatial and temporal enhancement capability to significantly augment low-photon voltage imaging.

**Approach:**

DeepVID v2 is built on our original DeepVID framework, which performs frame-based denoising by utilizing a sequence of frames around the central frame targeted for denoising to leverage temporal information and ensure consistency. Similar to DeepVID, the network further integrates multiple blind pixels in the central frame to enrich the learning of local spatial information. In addition, DeepVID v2 introduces a new spatial prior extraction branch to capture fine structural details to learn high spatial resolution information. Two variants of DeepVID v2 are introduced to meet specific denoising needs: an online version tailored for real-time inference with a limited number of frames and an offline version designed to leverage the full dataset, achieving optimal temporal and spatial performances.

**Results:**

We demonstrate that DeepVID v2 is able to overcome the trade-off between spatial and temporal performances and achieve superior denoising capability in resolving both high-resolution spatial structures and rapid temporal neuronal activities. We further show that DeepVID v2 can generalize to different imaging conditions, including time-series measurements with various signal-to-noise ratios and extreme low-photon conditions.

**Conclusions:**

Our results underscore DeepVID v2 as a promising tool for enhancing voltage imaging. This framework has the potential to generalize to other low-photon imaging modalities and greatly facilitate the study of neuronal activities in the brain.

## 1 Introduction

Voltage imaging is a powerful tool for studying the dynamics of neuronal activities in the brain. It enables the visualization of the spatiotemporal patterns of membrane potential changes in neurons, which is critical for understanding the underlying mechanisms of brain functions.^1^^,^^2^ Recently, two-photon imaging has also been adapted for voltage imaging, as it provides high spatial resolution and deep tissue penetration.^3^^–^^5^ However, voltage imaging data are often corrupted by strong noise, which hinders the accurate extraction of neuronal activities. The noise in voltage imaging data is mainly attributed to the low photon count of the fluorescence signal, which is further exacerbated by the high-speed acquisition required for capturing fast neuronal activities. The noise in voltage imaging data is often non-Gaussian and dominated by Poisson distribution, which poses a significant challenge for denoising.^6^

Deep learning–based denoising methods have shown great potential in addressing the challenges of denoising voltage imaging data. These methods have demonstrated superior performance in denoising various types of microscopy data, including fluorescence microscopy,^7^ light-sheet microscopy,^8^ and two-photon microscopy.^9^ However, in realistic denoising applications, the ground-truth high signal-to-noise ratio (SNR) measurements are often not available, which makes supervised learning–based methods less practical. In contrast, self-supervised learning–based methods have emerged as a promising alternative for denoising calcium or voltage imaging data, such as Noise2Void,^10^ DeepInterpolation,^11^ DeepVID,^3^^,^^12^ DeepCAD (and DeepCAD-RT),^13^^,^^14^ and SUPPORT.^15^ These self-supervised learning frameworks leverage the inherent spatial and/or temporal structure within the data, learning meaningful latent representations to perform denoising. With specifically designed tasks and loss functions, these models are adept at predicting a subset of data using the rest, bypassing the need for explicit supervision from ground-truth labels. This adaptability underscores their potential for robust denoising performance in applications with limited high-SNR data availability.

In voltage imaging, achieving high spatial resolution is essential for accurately resolving fine neuronal structures, whereas superior temporal resolution is crucial for capturing the rapid dynamics of neuronal activities. Traditional deep learning–based methods for denoising voltage imaging data often face a significant trade-off between spatial and temporal resolutions. Existing self-supervised learning frameworks typically require a large number of input frames,^11^^,^^14^ which leads to over-smoothed temporal traces and poor temporal resolution, or they use too few frames, resulting in low spatial resolution.^3^^,^^15^ Therefore, there is an unmet need for an advanced self-supervised denoising framework that can effectively decouple the spatial and temporal performances and achieve superior denoising capability in resolving both fine spatial structures and rapid temporal dynamics.

**Fig. 1:**
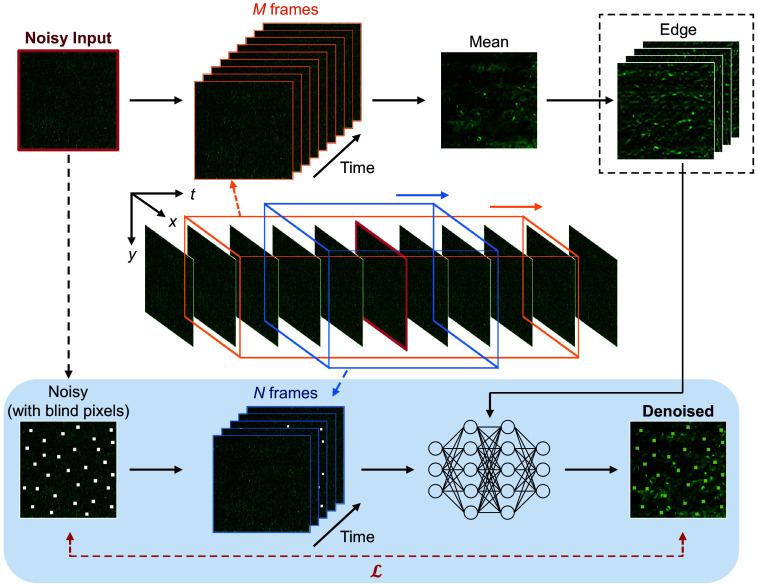
Block diagram of DeepVID v2. DeepVID v2 is composed of two main components: a main branch for denoising (bottom) and a side branch for spatial prior extraction (top). The edge extraction block (dotted square) in the side branch is applied in DeepVID v2-E while skipped in DeepVID v2-M. Components adapted from our original DeepVID network are represented in the blue-shaded area.

In this work, we present DeepVID v2, a self-supervised denoising framework with decoupled spatiotemporal enhancement for low-photon voltage imaging. In our previous work,^3^^,^^12^ we introduced DeepVID, which performs frame-based voltage imaging denoising by utilizing a sequence of frames around the target frame. This leverages temporal information while ensuring reconstruction consistency. Similar to DeepVID, the network also integrates multiple blind pixels in the target frame to enrich learning the local spatial information. To further enhance the spatial performance, DeepVID v2 presents a novel method to preserve fine structure information inherent in the raw data. Prior studies have demonstrated the effectiveness of utilizing edge as spatial prior information to improve the spatial resolution of denoised images.^16^^–^^18^ Here, by integrating an additional spatial prior extraction branch into the DeepVID network (Fig. 1), DeepVID v2 significantly enhances the spatial resolution and integrity of the neuronal structures in the denoised images.

We introduce two variants of DeepVID v2 to address the requirements of both online real-time and offline denoising, each capable of simultaneously recovering fast temporal dynamics and fine spatial structures. DeepVID v2-E leverages edge information as a spatial prior, achieving high performance with as few as 31 frames, making it potentially suited for real-time denoising applications. In contrast, DeepVID v2-M uses the mean frame of a long time-series measurement as the spatial prior, delivering optimal performance when accessing the entire dataset, making it ideal for offline denoising and analysis.

Critically, DeepVID v2 achieves the decoupling of spatial and temporal performances by introducing two adjustable parameters: the number of input frames, N, and the number of frames used for edge extraction, M. This dual-parameter strategy enables precise fine-tuning of the denoising process, allowing for optimal resolution of both spatial structures and temporal activities, thereby overcoming the limitations observed in previous models.

We demonstrate that DeepVID v2 achieves superior spatial and temporal denoising performances under diverse imaging conditions, including various SNRs and in extreme low-photon scenarios. Our results indicate that DeepVID v2 is a promising tool for denoising *in vivo* voltage imaging data, and has the potential to facilitate the study of neuronal activities in the brain.

## 2 Methods

### 2.1 Voltage Imaging Data Collection

The data used in this study are a two-photon voltage imaging image series collected from the spatiotemporal multiplexed ultrafast resonance frame-scanning (SMURF) microscope in our previous study.^3^ Spatial and temporal beam multiplexing along with a multianode photomultiplier tube (MAPMT) were used in the SMURF microscope setup. This configuration is engineered to maximize the effective repetition rate of pulsed lasers with minimal crosstalk on MAPMT, therefore enabling high-speed low-light imaging across a wide field of view (FOV). To measure the sensory-evoked neuronal responses, voltage imaging was performed at a sampling rate of 803 Hz in the primary somatosensory cortex (S1) from awake, head-fixed mice. Whisker stimulation was delivered as air puffs to the whisker pad at 10-Hz stimulus frequency in one- or five-puff trains, with 4-s intervals. The captured voltage imaging images contain 400×192  pixels in total concatenated from eight strips (400×24  pixels per strip), with a pixel size of 1.0  μm along the x-axis and 2.1  μm along the y-axis.

### 2.2 DeepVID v2 Framework

The system diagram of DeepVID v2 is illustrated in Fig. 1. DeepVID v2 performs denoising on three-dimensional (two-dimensional space + one-dimensional time) image stacks on a frame-by-frame basis. The network is composed of two main components: a main branch for denoising similar to our previously developed DeepVID^3^ and a side branch for spatial prior extraction. The edge extraction block (dotted square) in the side branch is applied only in DeepVID v2-E while skipped in DeepVID v2-M.

DeepVID v2 utilizes both the spatial and temporal information in the raw data, as well as additional spatial prior information from the side branch, to perform denoising. The neural network in the main branch is composed of four residual blocks, each containing two convolutional layers with batch normalization layers, followed by a parametric rectified linear unit (PReLU) activation layer attached after the first convolution layer. A skip connection is added between the input and output for each residual block (see details of network architecture in Fig. S1 in the Supplementary Material).

Given a frame to be denoised, the side branch first takes a $M=2M_{0}+1$ image series as the input, from $M_{0}$ frames before to $M_{0}$ frames after the central frame, to calculate a local mean frame, resulting in an improved spatial representation than any raw single frame. In DeepVID v2-M, the mean frame is directly fed into the main branch as an additional channel. In DeepVID v2-E, a Gaussian blur filter is then applied to the mean frame to remove residual noise, followed by four Sobel filters from 0, 45, 90, and 135 deg to extract the edge information along different directions. The outputs from the Sobel filters are then treated as four additional input channels to the main branch.

In addition to the spatial prior channels from the side branch, the main branch takes another $N=2N_{0}+1$ image series as the input, consisting of $N_{0}$ frames before and $N_{0}$ frames after the central frame, as well as the degraded central frame to perform denoising. A random set of pixels is set as blind pixels in the degraded central frame with a ratio of $p_{\text{blind}}$, whose intensities are replaced by random values sampled from the pixel intensities within the frame. These blind pixels are used to guide the network to learn the spatial and temporal information in the raw data and to prevent the network from simply replicating the input to the output.^3^

The loss function is the mean squared error (MSE) computed between the output denoised image and the input noisy image, calculated only at the locations of the blind pixels. In this study, parameters are optimized to achieve the best spatial and temporal performances at the same time, using seven frames as N, all available frames as M, and $p_{\text{blind}}=0.5\%$ for both DeepVID v2-E and DeepVID v2-M models. In consideration of real-time applications, M can be lowered to 31 frames with comparable spatial and temporal performances for DeepVID v2-E (see details in Sec. 4).

The training dataset comprises 1181 videos, with each video containing 1000 frames captured at a rate of 803 Hz. The training utilizes the Adam optimizer with a configuration of 360 steps per epoch and a batch size of four. To avoid overfitting, the training stopped after iterating through the entire dataset three times. The initial learning rate is set to $5\times 10^{-6}$, then halved if the loss on the validation set plateaus over the last 288,000 samples, until it reaches the minimum learning rate of $1\times 10^{-7}$.

### 2.3 Spike Detection

Spike detection is performed to infer evoked potentials from the extracted time traces. Slow fluctuation is first removed by baseline subtraction through a moving average of 2.5 s window. Time traces are then normalized by the mean of absolute values of the entire time trace. For each stimulus, spikes are detected in a window from 0 to 0.1 s after the stimulus onset, using a threshold of 4 and a minimum distance of 0.1 s between spikes. The full width at half maximum (FWHM) of the detected spikes is calculated as the time difference between the two points where the intensity reached half of the peak value. Only spikes with an FWHM falling within three standard deviations are retained. The number of detected spikes and the FWHM of these spikes are used to evaluate the temporal performance of the denoised videos.

### 2.4 Performance Metrics

The performance of DeepVID v2 is evaluated using a combination of reference-free and reference-based metrics that assess both temporal and spatial characteristics.

For temporal evaluation, the temporal SNR is defined as the ratio of the power of signal segments to the power of noise. Signal segments are identified as regions within the width of spikes detected from raw or denoised traces using any benchmark method. Given the sparse nature of the signals, the mean power of the entire trace is used as an estimate of the noise power.

Spatial performance is assessed through spatial resolution and spatial SNR, derived from the power spectral density (PSD) of individual frames. Spatial resolution is estimated based on the cutoff frequency, identified as the first point where the PSD curve intersects the estimated noise floor. Spatial SNR is calculated as the ratio of the power in the signal band to the power in the noise band, with the signal and noise bands defined as frequencies below and above the cutoff frequency, respectively.

In addition, the reference-based metrics, Pearson correlation coefficients (PCCs), are used to further evaluate the spatial and temporal performances. Spatial PCC is determined by calculating the pixel-wise correlation between the reference frame and the raw or denoised frame, with the temporal average frame serving as the reference. Temporal PCC is computed by correlating the reference time traces with the raw or denoised time traces, where the reference time traces are generated using a seven-frame moving average of the raw traces, matching the number of input frames used in DeepVID v2.

### 2.5 Dataset Division Based on Temporal SNR

The dataset is divided into two subsets representing low and high SNRs. The averaged temporal SNR of each FOV is obtained by averaging the temporal SNR calculated from each region of interest (ROI) trace for all the ROIs in the FOV. The FOVs are then sorted by the averaged temporal SNR. The bottom and top halves are grouped as the low and high SNR subsets, respectively.

### 2.6 Simulation of Videos in Lower-Photon Regimes

**Fig. 2:**
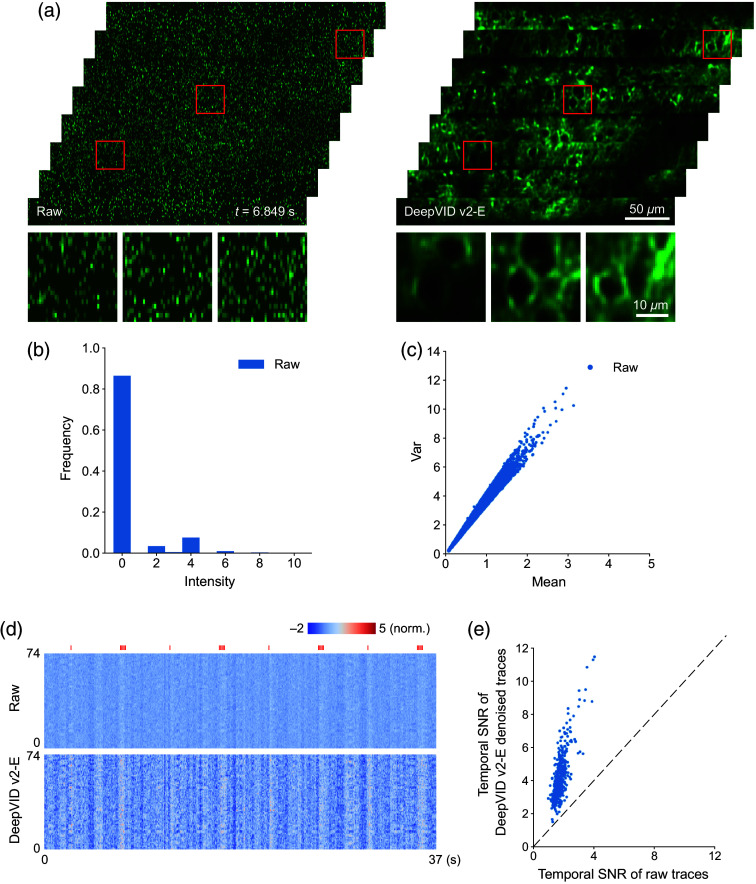
DeepVID v2 denoising enhances both the spatial and temporal quality of the voltage imaging data. (a) Single-frame images from the raw and DeepVID v2-E–denoised videos. Zoomed-in images of each region outlined on the top are shown at the bottom. (b) Histogram of the raw video. (c) Characteristics of noise in the raw video. The variance of single-pixel time traces (Y-axis) is linearly proportional to the mean of the traces (X-axis). (d) Heatmaps displaying time traces extracted from 74 ROIs in the raw and DeepVID v2-E–denoised videos. Air puff whisker stimuli are shown as red ticks on the top. (e) Temporal SNRs of the raw and DeepVID v2-E–denoised time traces.

In low-photon regimes, the signal is dominated by Poisson noise, in which the variance is proportional to the mean intensity. This feature is verified in our dataset by calculating the mean and variance of each single-pixel time trace [Fig. 2(c)]. The ratio of this linear correlation, β, reflects the characteristic of the imaging system and therefore should be stable in varied light conditions. The simulated video in lower-photon conditions should follow the same principle, whose variance is still proportional to the mean intensity, with the same ratio as the raw video.

To simulate voltage imaging data in lower photon regimes, we propose a two-step simulation protocol on a pixel-by-pixel basis. Before the simulation, we calculate the ratio $\beta_{0}$ between the variance and the mean intensity for the raw video.

First, for each pixel intensity in the raw video $I_{0}$, we apply binomial degradation with a probability of p to obtain $I_{b}$

$$I_{b}∼\text{Binomial}(I_{0},p).$$

We calculate the updated ratio $\beta_{b}$ after applying binomial degradation to all pixels in the video. This step reduces the intensity of the measurements but also lowers the ratio.

Second, we multiply all pixel intensities in the simulated video $I_{b}$ by a factor of $A=\beta_{0}/\beta_{b}$ to obtain $I_{d}$, which increases both the intensity and the ratio by a factor of A. After this two-step simulation, the simulated video $I_{d}$ has a lower intensity with a factor of d=pA compared with the raw video $I_{0}$, whereas the ratio between the variance and the mean remains the same (Fig. S14 in the Supplementary Material). The proposed simulation protocol is able to simulate voltage imaging data in lower photon regimes while maintaining the same characteristics of the imaging system.

## 3 Results

### 3.1 DeepVID v2 Improves Spatial Resolution While Preserving Temporal Dynamics

To demonstrate the denoising capability of DeepVID v2, we present single-frame full-FOV images from both the raw and DeepVID v2-E–denoised videos in Fig. 2(a). The noisy raw video was captured in an extremely low-photon regime, with the raw pixel intensity readout lower than 10 for almost all pixels [Fig. 2(b)]. The variance of single-pixel time traces is linear to the mean of such traces, which validates that Poisson noise dominates the raw measurements [Fig. 2(c)]. After denoising, the membrane and other neuronal structures are clearly resolved at the single-frame level. Heatmaps displaying time traces extracted from 74 manually labeled ROIs with active neurons during the 37-s measurements are depicted in Fig. 2(d) to highlight the improvement from the DeepVID v2 denoising. The heatmap of traces from the denoised video exhibits a more pronounced contrast compared with the raw video, suggesting enhanced signals from the underlying neuronal activities after denoising. We calculate the temporal SNRs for the raw and DeepVID v2-E–denoised time traces extracted from all ROIs in the FOV, as presented in Fig. 2(e). The temporal SNRs of denoised time traces are consistently higher than that of the raw traces for all ROIs (DeepVID v2-E, 4.19±1.33; raw, 1.75±0.37; n=516), further underscoring the effective temporal denoising capability of DeepVID v2.

**Fig. 3:**
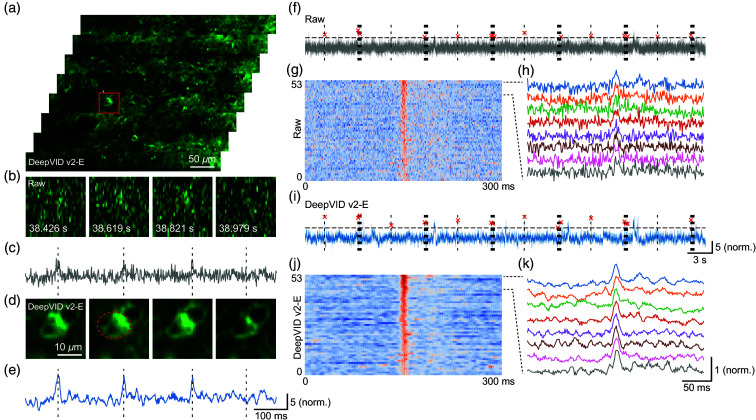
Denoising performance on single-neuron activities. (a) Single-frame full-FOV denoised image. (b) Zoomed-in view and (c) time trace of the ROI from the raw video. (d) Zoomed-in view and (e) time trace of the ROI from the DeepVID v2-E–denoised video. (f) Detected evoked potentials. (g) Heatmap of the detected evoked potentials. (h) Time traces of the detected evoked potentials from the raw video. (i) Detected evoked potentials. (j) Heatmap of the detected evoked potentials. (k) Time traces of the detected evoked potentials from the DeepVID v2-E–denoised video. Air puff whisker stimuli are shown as dotted lines, and detected evoked potentials are shown as red crosses in panels (f) and (i).

Next, we investigate the performance of DeepVID v2 with a focus on single-neuron activities. From another time-series measurement with a single-frame full-FOV denoised image shown in Fig. 3(a), we extract a few key frames from an ROI in Fig. 3(d), along with the corresponding raw frames in Fig. 3(b). The time traces from an active neuron [circled in red in Fig. 3(d)] are extracted from both the raw and denoised videos, as shown in Figs. 3(c) and 3(e), respectively. The activation on the neuronal membranes is consistently resolved in the zoomed-in images at the timestamps marked on the time traces.

We further apply spike detection on the time traces to extract evoked potentials (Fig. S2 in the Supplementary Material). The evoked potentials extracted are marked in red crosses, whereas the stimuli are shown as dotted lines in Figs. 3(f) and 3(i). All 45 detected evoked potentials are aligned at the peak and presented as heatmaps in Figs. 3(g) and 3(j). The time traces of the evoked potential are displayed in Figs. 3(h) and 3(k). The evoked potentials extracted from the denoised video exhibit less noisy traces compared with the raw video, which indicates the improved capability of DeepVID v2 in resolving neuronal activities.

### 3.2 DeepVID v2 Overcomes Trade-off Between Spatial and Temporal Performances

The performance of previous self-supervised denoising algorithms has often been influenced by the trade-off between spatial and temporal resolutions, which is controlled by the number of input frames N to the network (Fig. 5 and Fig. S11 in the Supplementary Material). Unlike previous methods, DeepVID v2 is designed to decouple spatial and temporal performances by incorporating two key parameters: the number of input frames, N, and the number of frames used for edge extraction, M. To investigate the effect of these parameters on the performance of DeepVID v2-E, we vary N and M and train a neural network model for each combination.

**Fig. 4:**
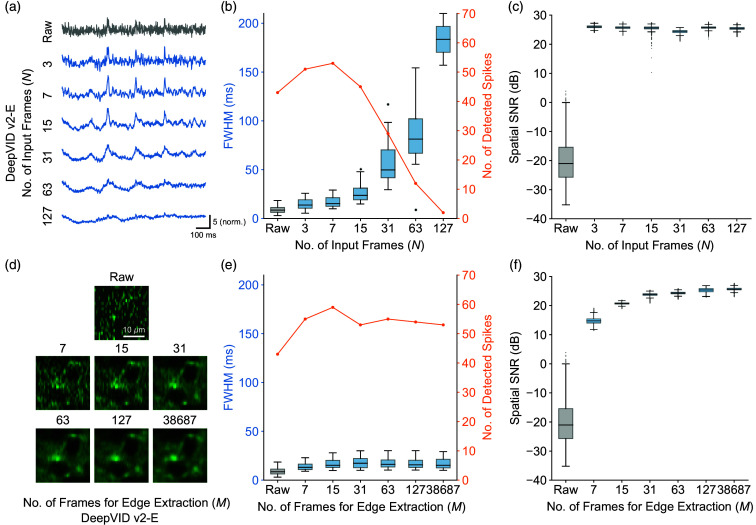
Parameter analysis. (a) Time traces extracted from the same ROI from the DeepVID v2-E–denoised videos with different N. (b) Temporal metrics based on spike detection. (c) Spatial SNR of the DeepVID v2-E–denoised videos with different N. (d) Zoomed-in view of an ROI from a single-frame image in the DeepVID v2-E–denoised videos with different M. (e) Temporal metrics based on spike detection. (f) Spatial SNR of the DeepVID v2-E–denoised videos with different M.

First, we fix M as the maximum available frames and vary N from 3 to 127. As N increases, DeepVID v2-E–denoised time traces become over-smoothed and spikes become harder to recognize, as shown in Fig. 4(a) and Fig. S3 in the Supplementary Material. To evaluate the temporal performance, spike detection is performed on the time traces, and the temporal metrics including the number of detected spikes and the FWHM of the detected spikes are calculated. The FWHM of the detected spikes increases with increasing N, whereas the number of detected spikes initially increased due to improved temporal SNR but later decreased as the traces become over-smoothed, as shown in Fig. 4(b) and Figs. S5(a) and S5(b) in the Supplementary Material. The spatial SNRs of the denoised videos are significantly higher than that of the raw video but remain comparable across different N, indicating that the spatial performance is not significantly affected by N, as shown in Fig. 4(c) and Figs. S4, S5(c), and S5(d) in the Supplementary Material. From this analysis, we conclude that the optimal value for N is 7 for our experimental conditions, which provides the best combination of narrow FWHMs and a large number of reliably detected spikes.

Next, we fix N at seven frames for the optimal temporal performance and vary M from seven to the maximum available frames. Figure 4(d) shows the zoomed-in views of an ROI from a single-frame image in the raw and denoised videos using DeepVID v2-E with different M indicated above each image. The number of detected spikes and the FWHM of the detected spikes are comparable across different M, indicating that the temporal performance is not significantly affected by M, as shown in Fig. 4(e) and Figs. S6, S7(a), and S7(b) in the Supplementary Material. The spatial SNR of the denoised videos increases with M at the beginning and then reaches a plateau from 31 frames, as shown in Fig. 4(f) and Figs. S7(c) and S7(d) in the Supplementary Material, suggesting better spatial performance with an increased M when M is small.

Our parameter analysis reveals that the new framework of DeepVID v2-E is able to decouple the spatial and temporal performances by independently adjusting M and N. This decoupling enables DeepVID v2 to achieve superior denoising capability in resolving both spatial neuronal structures and temporal neuronal activities with high SNR.

For the DeepVID v2-M model, the decoupling of the spatial and temporal performances still exists when M is large, allowing the best temporal and spatial performances to be achieved simultaneously (Figs. S8 and S9 in the Supplementary Material). However, in a similar scenario, while fixing N at seven frames, the time traces extracted from the DeepVID v2-M–denoised videos significantly deteriorate as M decreases, as shown in Fig. S10 in the Supplementary Material. The best performance is achieved only when M is set to the maximum number of available frames in the measurement. At M=31 frames, where the DeepVID v2-E model nearly achieves its optimal performance, the DeepVID v2-M model still suffers from poor spatial and temporal performances. This suggests that the DeepVID v2-M model is better suited for offline processing tasks when the entire time-series measurements are available for spatial prior extraction.

**Fig. 5:**
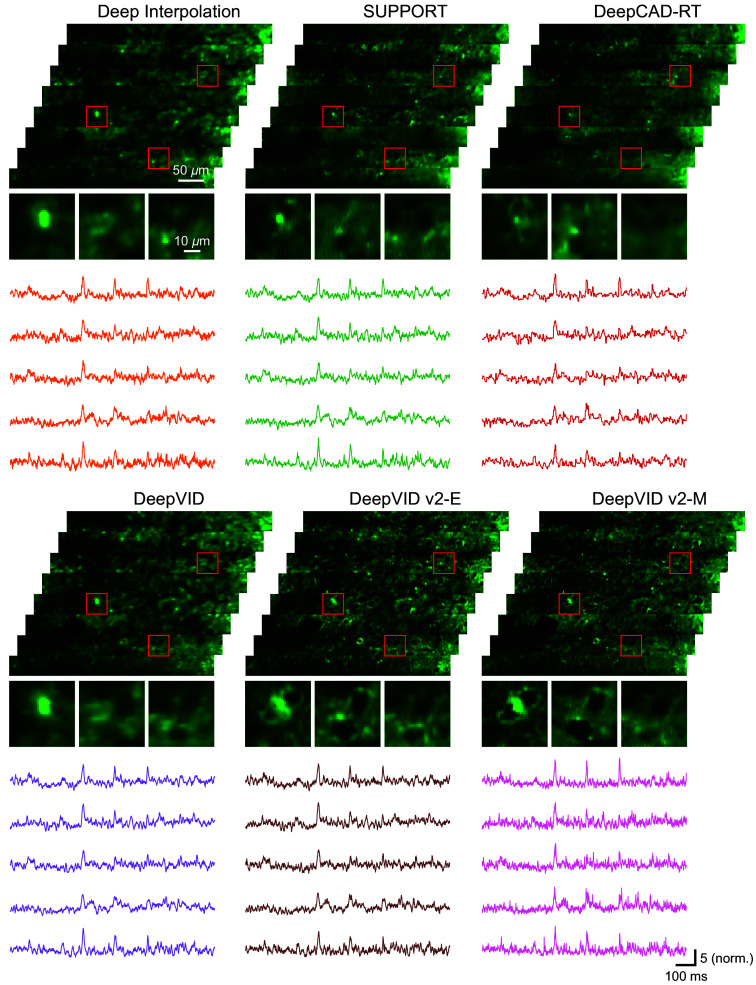
Qualitative visualization of benchmark comparison. Single-frame images and ROI time traces from the raw and denoised videos. All benchmarks utilize N=7 frames as input to the network. Both DeepVID v2-E and DeepVID v2-M maintain the optimal parameter settings with N=7 and M using all available frames, due to the advantage of two adjustable parameters.

**Fig. 6:**
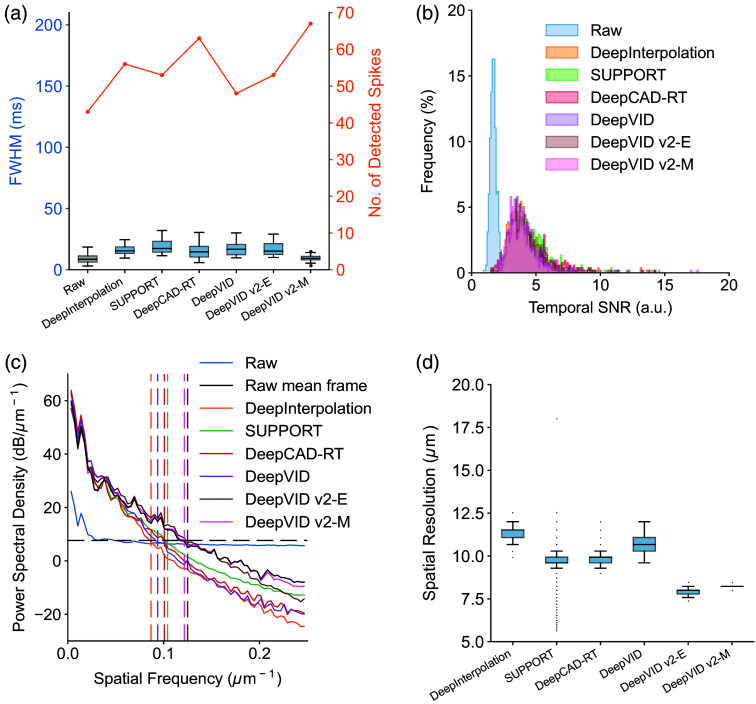
Quantitative evaluation of benchmark comparisons. (a) Number of detected spikes and FWHM of the detected spikes from the raw and denoised ROI time traces. (b) Histogram of temporal SNR for raw and denoised ROI time traces. (c) PSD of single frames in raw and denoised videos averaged across frames. (d) Spatial resolution of single frames in raw and denoised videos. All benchmarks use N=7 frames as input to the network. Both DeepVID v2-E and DeepVID v2-M maintain optimal performance with N=7 and M set to use all available frames, benefiting from the flexibility of two adjustable parameters.

We further conduct a comparative analysis of DeepVID v2 against other recently developed self-supervised denoising methods, including DeepInterpolation,^11^ SUPPORT,^15^ DeepCAD-RT,^14^ and our previously developed DeepVID.^3^^,^^12^ We compare the performance of all benchmark networks in two conditions, one with a small N at 7 frames (Figs. 5 and 6) and another with a large N at 127 frames (Figs. S11 and S12 in the Supplementary Material). Both DeepVID v2-E and DeepVID v2-M maintain the same optimal parameter settings due to the advantage of two adjustable parameters, with N=7 and M using all available frames. Our qualitative evaluation focuses on spatial performance using single-frame images and temporal performance using time traces extracted from ROIs. For quantitative metrics, we conduct spike detection to calculate spike statistics and temporal SNR for temporal evaluation and compute spatial resolution derived from spatial PSD for spatial evaluation.

In the first comparison, all benchmarks utilize N=7 frames as input to the network. DeepVID v2-E shows comparable temporal performance with other benchmarks, demonstrating a similar number of detected spikes, FWHM of the detected spikes, and temporal SNR [Figs. 5, 6(a), and 6(b)]. DeepVID v2-M achieves the highest temporal performance, characterized by the greatest spike heights, narrowest spike widths, and highest spike counts. Notably, DeepVID v2-M is the only method that achieves spike widths similar to those of the raw data, whereas spike widths from other benchmarks are consistently larger than those of the raw data. Jagged edges in the traces are observed only in DeepCAD-RT, which struggles with robust temporal convolution due to the limited number of input frames. For spatial performance, both DeepVID v2-E and DeepVID v2-M exhibit clear spatial structures with significantly lower spatial resolution [Figs. 5, 6(c), and 6(d)], outperforming all other benchmarks that suffer from blurring in single frames. SUPPORT demonstrates relatively good but inconsistent spatial performance, showing clear structures in regions of high spatial SNR, but significant blurring in areas with lower spatial SNR.

In the second comparison, all benchmarks, except for DeepVID v2-E and DeepVID v2-M, utilize N=127 frames as input to the network. Although all benchmarks exhibit similar spatial performance with clear structures, only DeepVID v2-E and DeepVID v2-M sustain optimal temporal performance, characterized by a consistent spike count and narrow FWHM of detected spikes (Figs. S11 and S12 in the Supplementary Material).

Except for DeepVID v2-E and DeepVID v2-M, all other benchmarks encounter a trade-off between spatial and temporal performances: achieving good spatial but poor temporal performance with small N (Figs. 5 and 6), and vice versa with large N (Figs. S11 and S12 in the Supplementary Material). By decoupling spatial and temporal performances into two parameters, DeepVID v2 successfully overcomes this trade-off, thereby achieving superior performance in both spatial and temporal metrics simultaneously.

### 3.3 DeepVID v2 Generalizes to Different Imaging Conditions

**Fig. 7:**
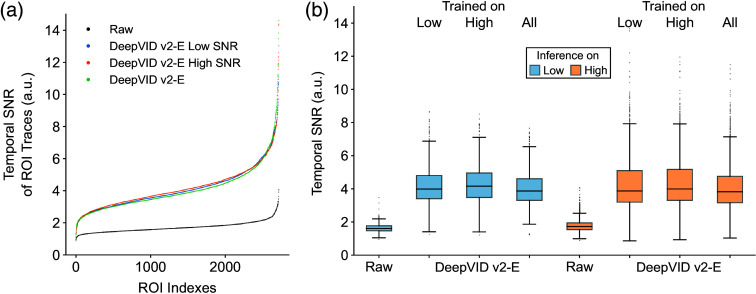
Generalization across measurements with varying temporal SNRs. (a) Temporal SNR of ROI time traces from raw and denoised videos for three DeepVID v2-E models, each trained on subsets with different temporal SNR levels. (b) Temporal SNR of ROI time traces from raw and denoised videos, grouped by SNR subsets used for inference (color) and the corresponding trained models (X-axis).

To evaluate the generalization capability of DeepVID v2 under different imaging conditions, we train and infer on voltage imaging data with various temporal SNRs. To perform this study, our experimental dataset is divided into two subsets, labeled as low and high SNR groups. The data division is based on the averaged temporal SNR calculated on all the manually labeled ROIs for each FOV in our dataset, as detailed in Sec. 2.5. A separate DeepVID v2-E model is trained for each subset. The temporal SNR of each ROI time trace after denoising using each model is calculated and presented in Fig. 7. The ROIs are sorted by the temporal SNR for each condition in Fig. 7(a). The temporal SNR of raw data spans a wide range from 0.89 to 4.07, as shown in the black curve. The temporal SNR after denoising by all three DeepVID v2-E models consistently improves by over 2.3-fold for all ROIs (low SNR model, 2.38±0.55; high SNR model, 2.41±0.48; all SNR model, 2.33±0.52; n=2717), regardless of the SNR of the raw video, indicating that DeepVID v2 is able to generalize to data with various temporal SNRs (see Fig. S13 in the Supplementary Material for single-frame images and time traces).

**Fig. 8:**
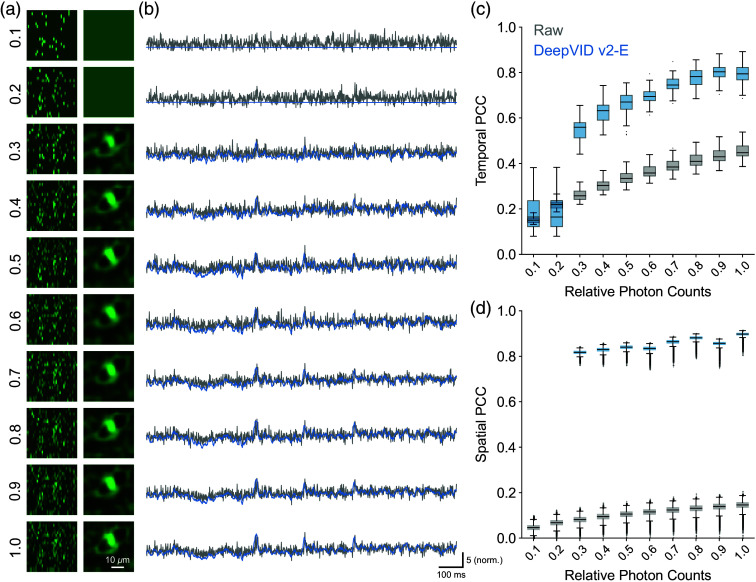
Simulation of DeepVID v2 denoising capability in extreme low-photon regimes. (a) Zoomed-in image of an ROI. (b) Time traces from the simulated noisy and denoised videos. (c) Temporal PCC of the simulated noisy and denoised time traces. (d) Spatial PCC of the simulated noisy and denoised videos.

We further evaluate DeepVID v2 in extreme low-photon regimes. To perform this study, we simulate voltage imaging data at various photon levels from 10% to 100% with respect to the original measurement (see details in Sec. 2.6 and Fig. S14 in the Supplementary Material) and test the denoising performance of DeepVID v2. For each photon level, we train a separate DeepVID v2-E model, which is possible as DeepVID v2 is a self-supervised method that does not need external ground-truth data for training. The zoomed-in image of an ROI and the time traces extracted from the simulated noisier measurements and the denoised videos are presented in Figs. 8(a) and 8(b), respectively. DeepVID v2-E is able to reliably perform denoising on data with photon levels as low as 30% of the original measurement. Both the temporal PCC [Fig. 8(c)] and spatial PCC [Fig. 8(d)] show dramatic improvements after denoising for photon levels as low as 30% of the original. The results underscore DeepVID v2’s ability to perform voltage imaging denoising in extreme low-photon conditions, which is critical for further pushing the limits in voltage imaging *in vivo*.

## 4 Discussion and Conclusion

We introduced DeepVID v2, a self-supervised denoising framework with decoupled spatiotemporal enhancement capabilities tailored for low-photon voltage imaging. By integrating an additional spatial prior extraction branch into the DeepVID architecture^3^ and incorporating two adjustable parameters, DeepVID v2 effectively addresses the inherent trade-off between spatial and temporal performances, enhancing its denoising capabilities for resolving both fine spatial neuronal structures and rapid temporal dynamics. Our experiments further demonstrated the robustness of DeepVID v2 across a range of imaging conditions, including varying SNR levels and extreme low-photon scenarios, underscoring its potential as a powerful tool for denoising voltage imaging data and advancing the study of neuronal activities within the brain.

We reported two variants of DeepVID v2: DeepVID v2-E and DeepVID v2-M, designed for online real-time denoising with limited frames and offline denoising with full access to the data, respectively, to achieve optimal temporal and spatial performances. DeepVID v2-M performs optimally with N=7 and M set to use all available frames, delivering the highest temporal performance with the greatest spike heights, narrowest spike widths, and detailed spatial reconstructions. However, its temporal performance degrades significantly when the number of frames is limited. Conversely, DeepVID v2-E achieves near-optimal temporal and spatial performances with as few as N=7 and M=31 frames, making it suitable for real-time denoising applications, although its performance is slightly lower compared with DeepVID v2-M.

However, neither variant can recover clean spatial structures when the number of frames used for spatial prior extraction is extremely low (e.g., N=3 and M=3), due to the limited SNR of the spatial prior. This reveals a persistent trade-off between performance and real-time capability (see Fig. S15 in the Supplementary Material), highlighting an area for future improvement.

A limitation of this approach is that the performance of DeepVID v2 can be affected by the relationship between imaging speed and object motion. DeepVID v2’s spatial prior extraction assumes that the local mean frame is free of motion artifacts, which may not be valid when the object’s movement is significantly faster than the imaging speed. In such cases, the SUPPORT framework offers an alternative solution, demonstrating its denoising capability on moving *C. elegans* when object locomotion exceeds imaging speed. SUPPORT relies more on spatial information from neighboring pixels in the central frame rather than temporal information from adjacent frames.^15^ However, the spatial performance of SUPPORT is sensitive to the raw data quality, as observed in the varying spatial performance across different strips in our benchmark comparison (Fig. 5). Achieving robust denoising in high-speed, low-photon, large-FOV imaging where object movement outpaces imaging speed remains challenging, necessitating further methodological advancements to effectively address this issue.

## Supplementary Material



## Data Availability

Code for PyTorch implementation of DeepVID v2 is available at the GitHub repository: https://github.com/bu-cisl/DeepVIDv2. Data used in this study are obtained from our previous publication.^3^
