# Supplementary material for: DeepVID v2: self-supervised denoising with decoupled spatiotemporal enhancement for low-photon voltage imaging
Source: Neurophotonics. 2024 Oct 29;11(4):045007. doi: 10.1117/1.NPh.11.4.045007 (PMC11519979; doi:10.1117/1.NPh.11.4.045007)
Supplement: Supplementary file 1 [file NPh_011_045007_SD001.pdf]

Supplementary Materials

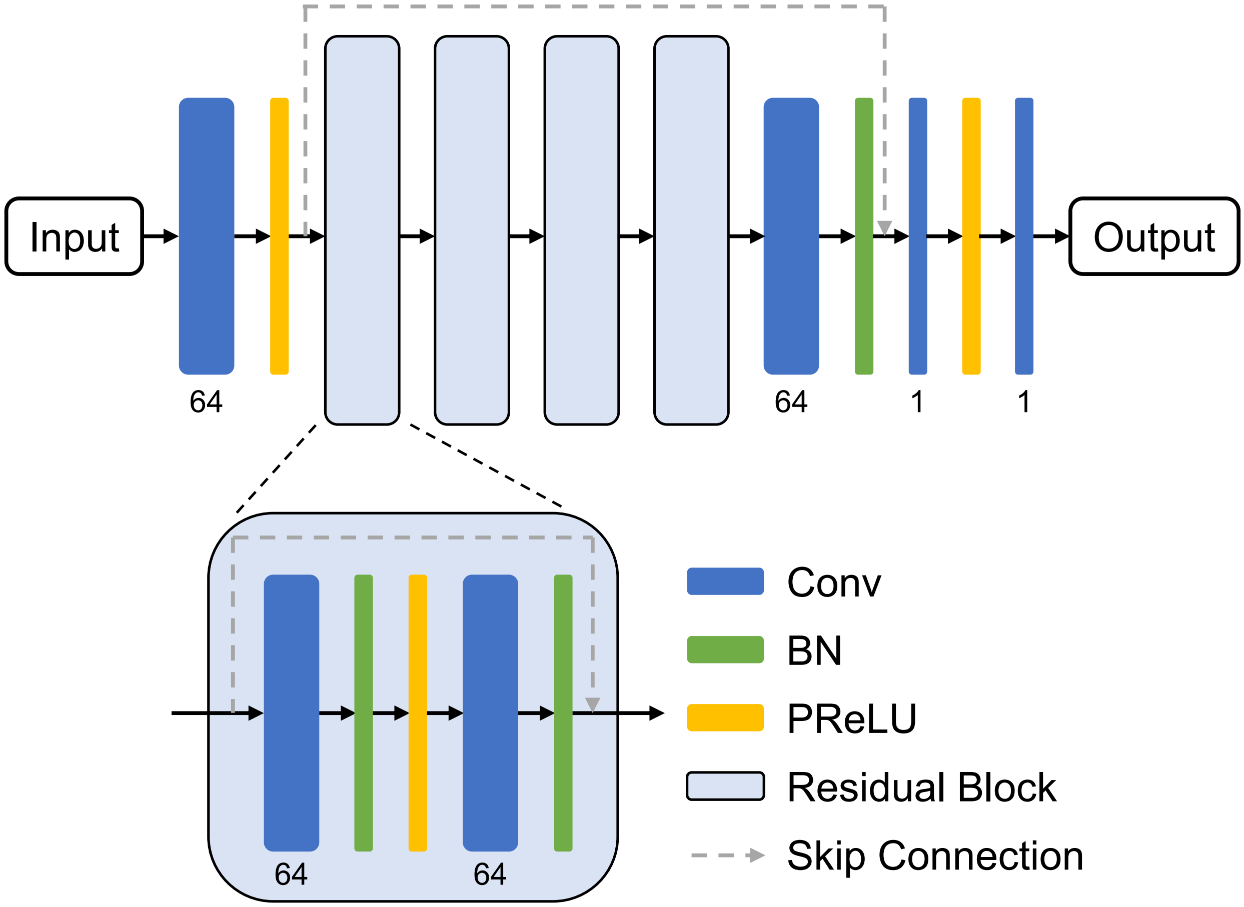

Supplementary Fig 1 Network architecture of the main network in DeepVID v2.

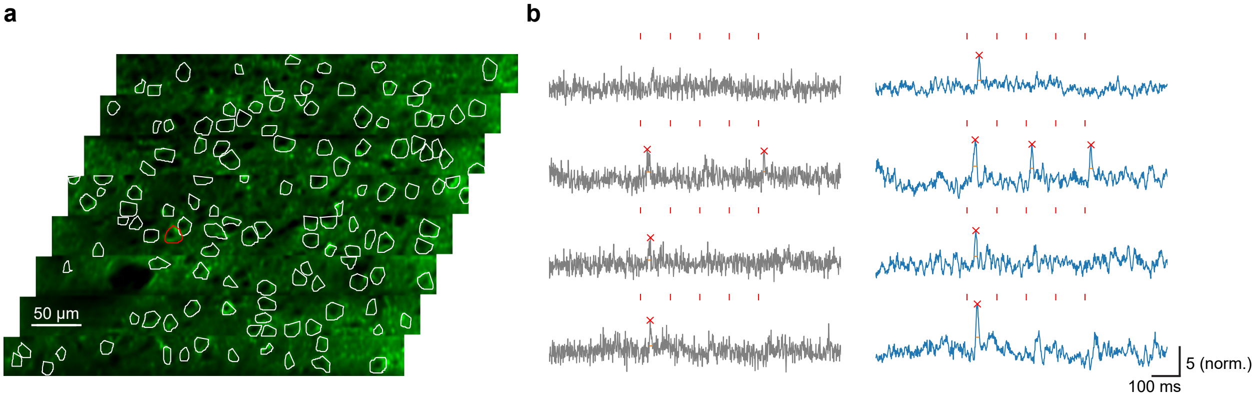

Supplementary Fig 2 (a) Manually labeled regions of interest (ROIs). (b) Example of spike detection results on the time trace of a ROI from the raw (left) and the DeepVID v2-E denoised video. Air puff whisker stimuli are shown as red ticks on the top.

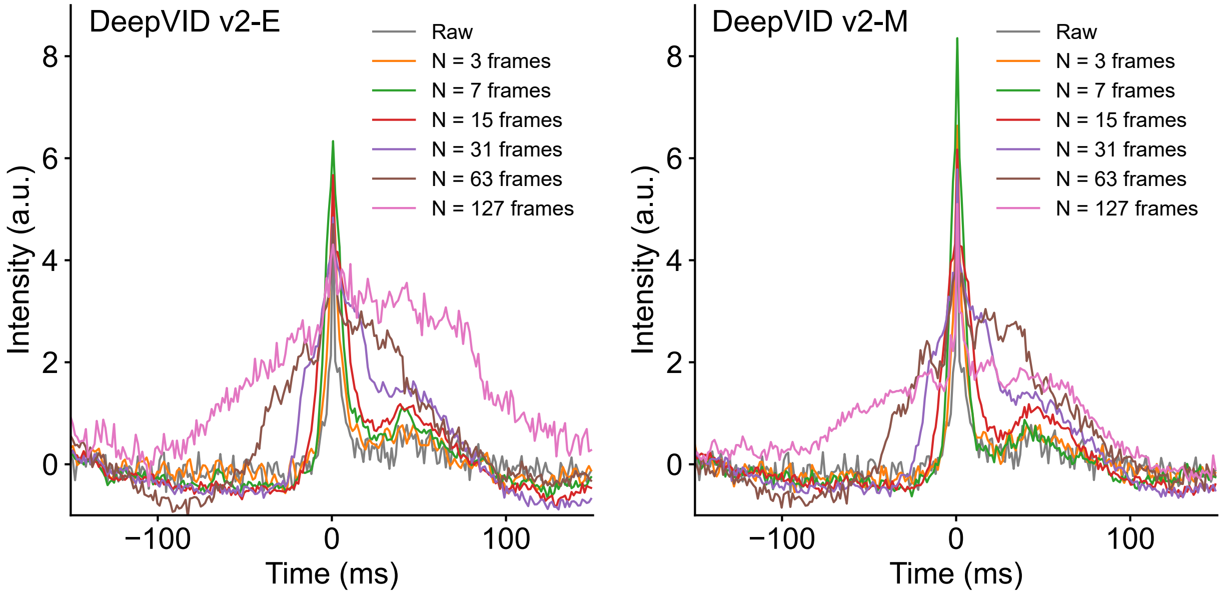

Supplementary Fig 3 Overlay of the mean time trace of a ROI for the raw and DeepVID v2-E and DeepVID v2-M denoised videos. The mean time trace is averaged from all detected spikes on a ROI for each condition. DeepVID v2-E and DeepVID v2-M models are trained with various N from 3 to 127 frames, and all available frames (M) for edge extraction.

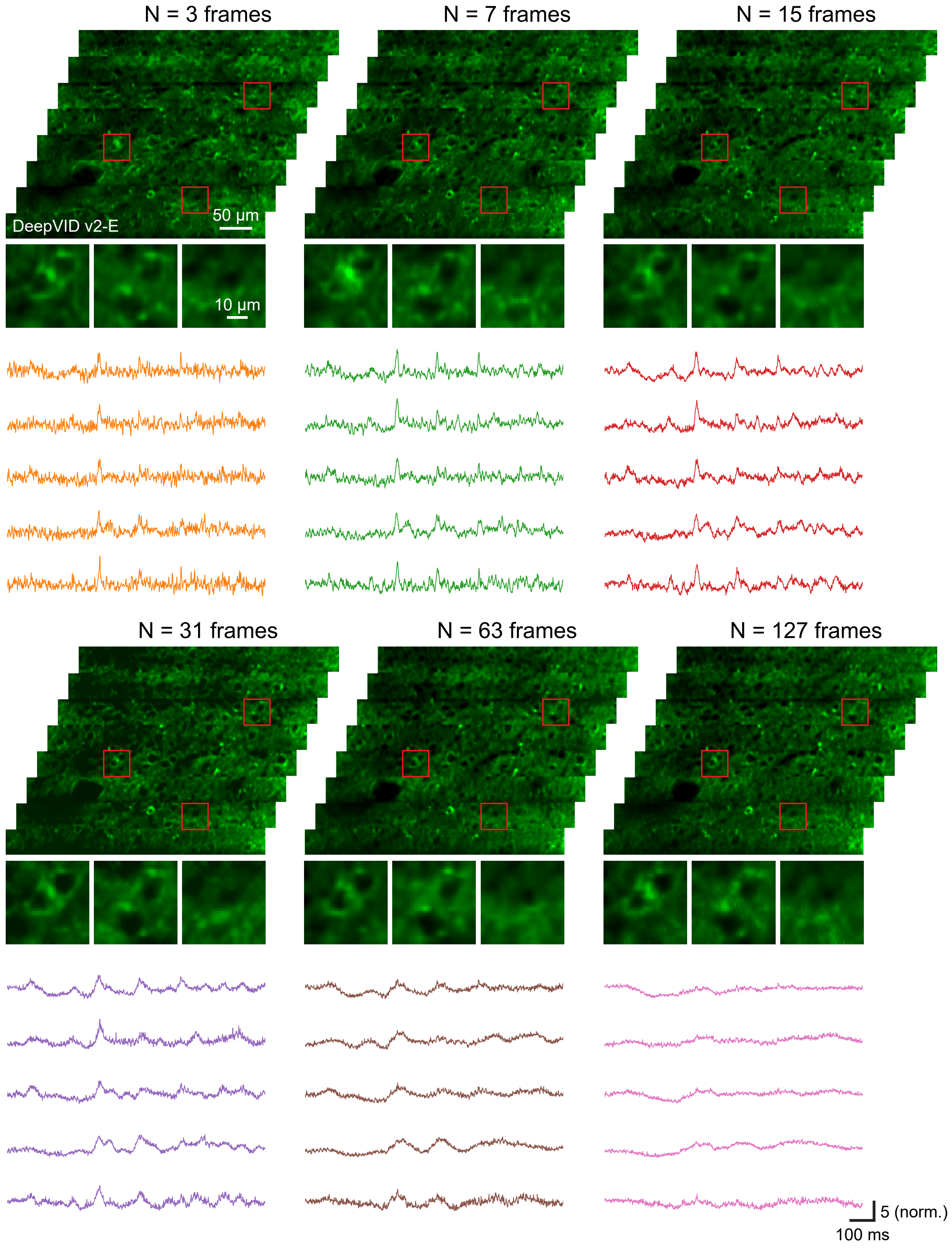

Supplementary Fig 4 Qualitative visualization of parameter analysis. Single-frame images and ROI time traces from DeepVID v2-E denoised videos trained with various input frames N from 3 to 127 frames, and all available frames for edge extraction (M).

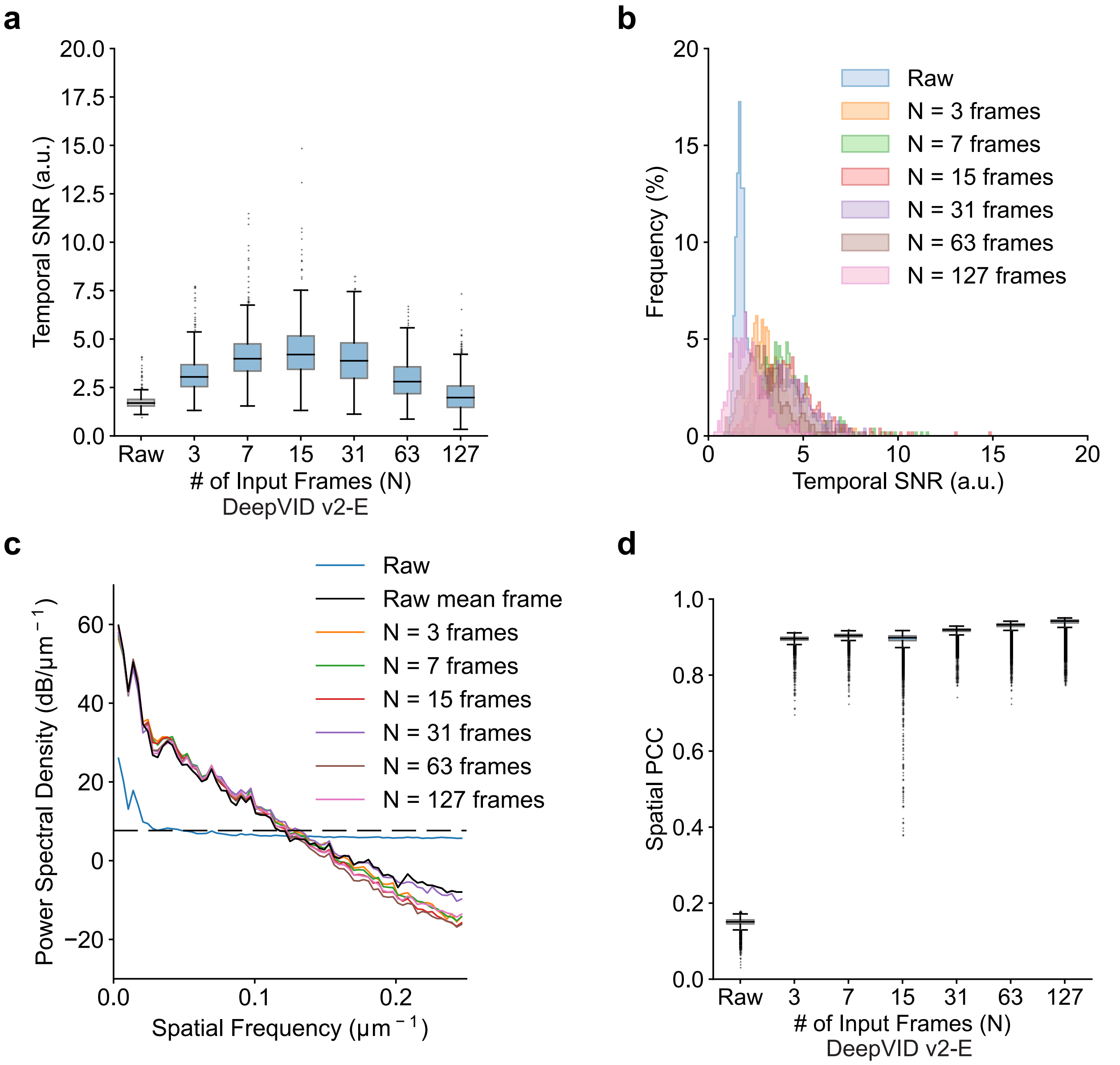

Supplementary Fig 5 Quantitative evaluation of parameter analysis. (a) The number of detected spikes and FWHM of the detected spikes from the raw and denoised ROI time traces. (b) The histogram of temporal SNR of raw and denoised ROI time traces. (c) Power spectral density from single frames in raw and denoised videos, averaged over frames. (d) Spatial PCC of single frames in raw and denoised videos. DeepVID v2-E trained with various input frames N from 3 to 127 frames, and all available frames for edge extraction (M).

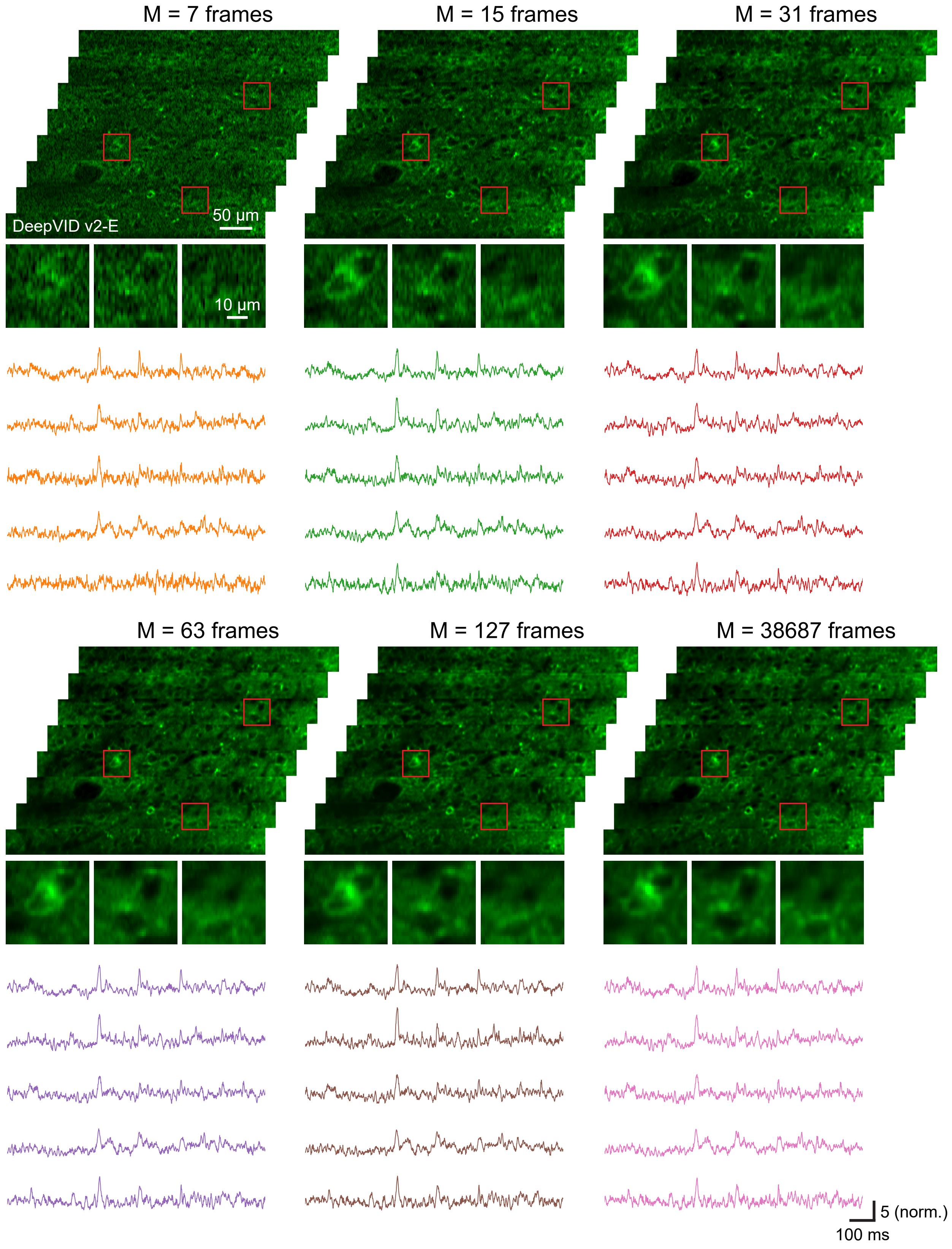

Supplementary Fig 6 Qualitative visualization of parameter analysis. Single-frame images and ROI time traces from DeepVID v2-E denoised videos trained with fixed input frames N at 7 frames, and various frames for edge extraction (M) from 7 to the maximum available frames.

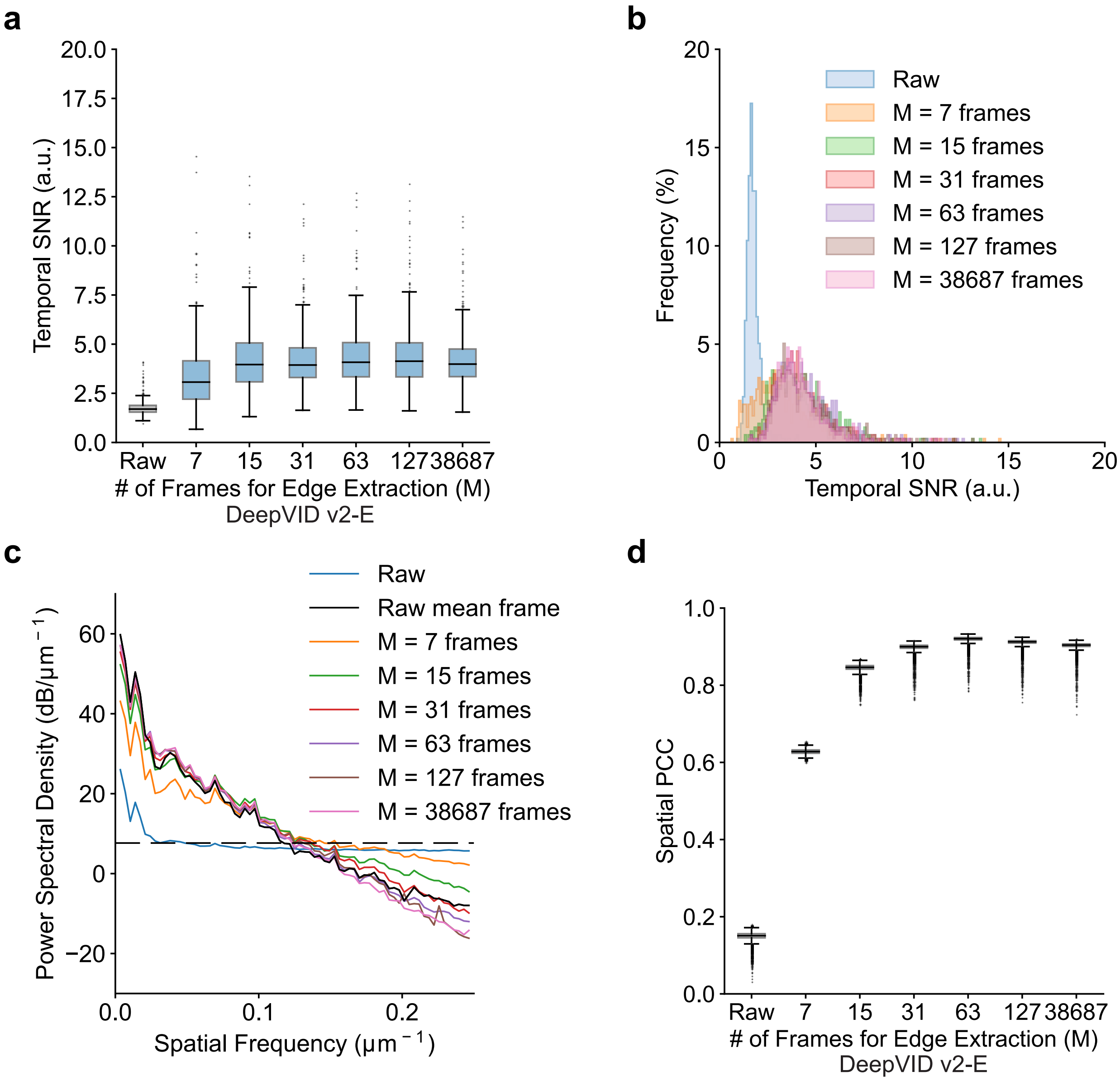

Supplementary Fig 7 Quantitative evaluation of parameter analysis. (a) The number of detected spikes and FWHM of the detected spikes from the raw and denoised ROI time traces. (b) The histogram of temporal SNR of raw and denoised ROI time traces. (c) Power spectral density from single frames in raw and denoised videos, averaged over frames. (d) Spatial PCC of single frames in raw and denoised videos.DeepVID v2-E trained with fixed input frames N at 7 frames, and various frames for edge extraction (M) from 7 to the maximum available frames.

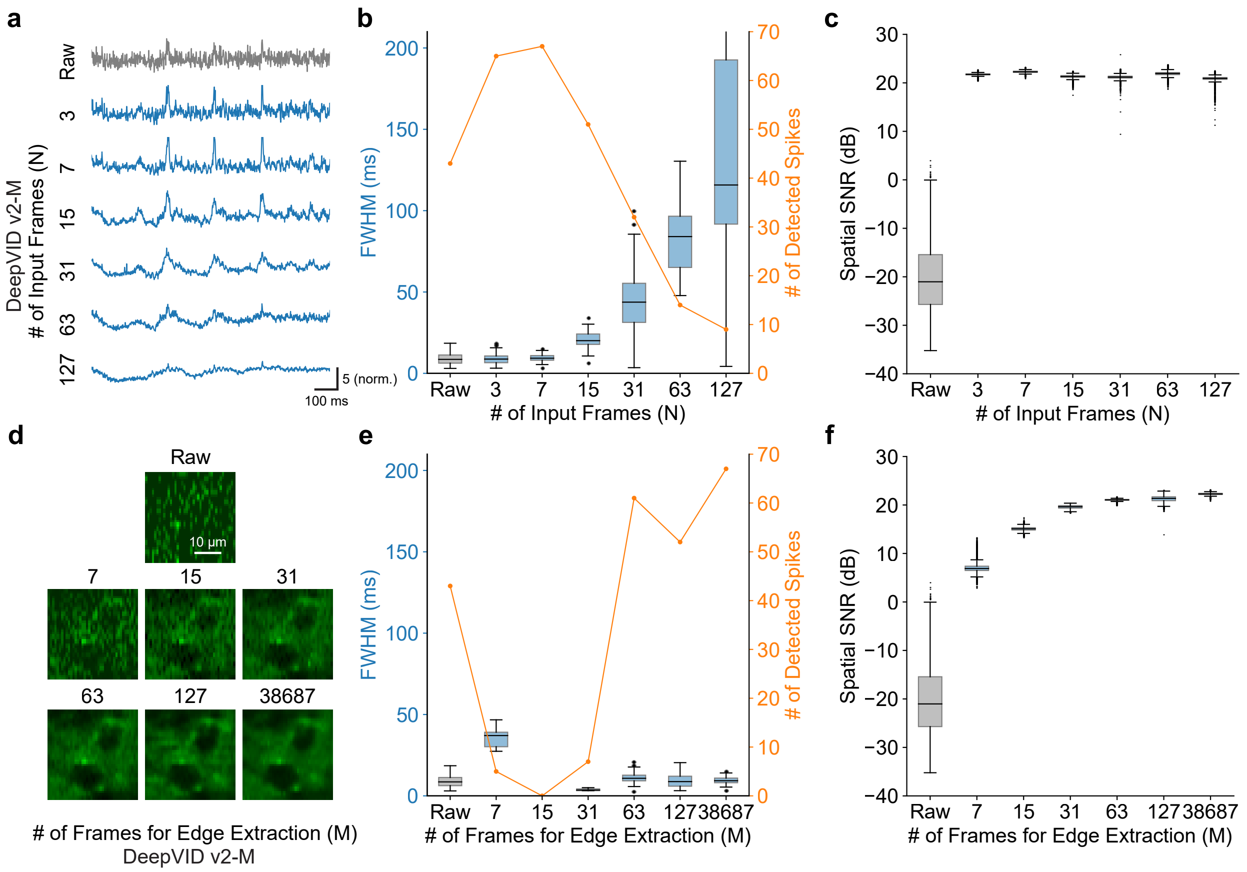

Supplementary Fig 8 Parameter analysis. (a) Time traces extracted from the same ROI from the DeepVID v2-M denoised videos with different N. (b) Temporal metrics based on spike detection and (c) spatial SNR of the DeepVID v2-M denoised videos with different N. (d) Zoom-in view of a ROI from a single-frame image in the DeepVID v2-M denoised videos with different M. (e) Temporal metrics based on spike detection and (f) spatial SNR of the DeepVID v2-M denoised videos with different M.

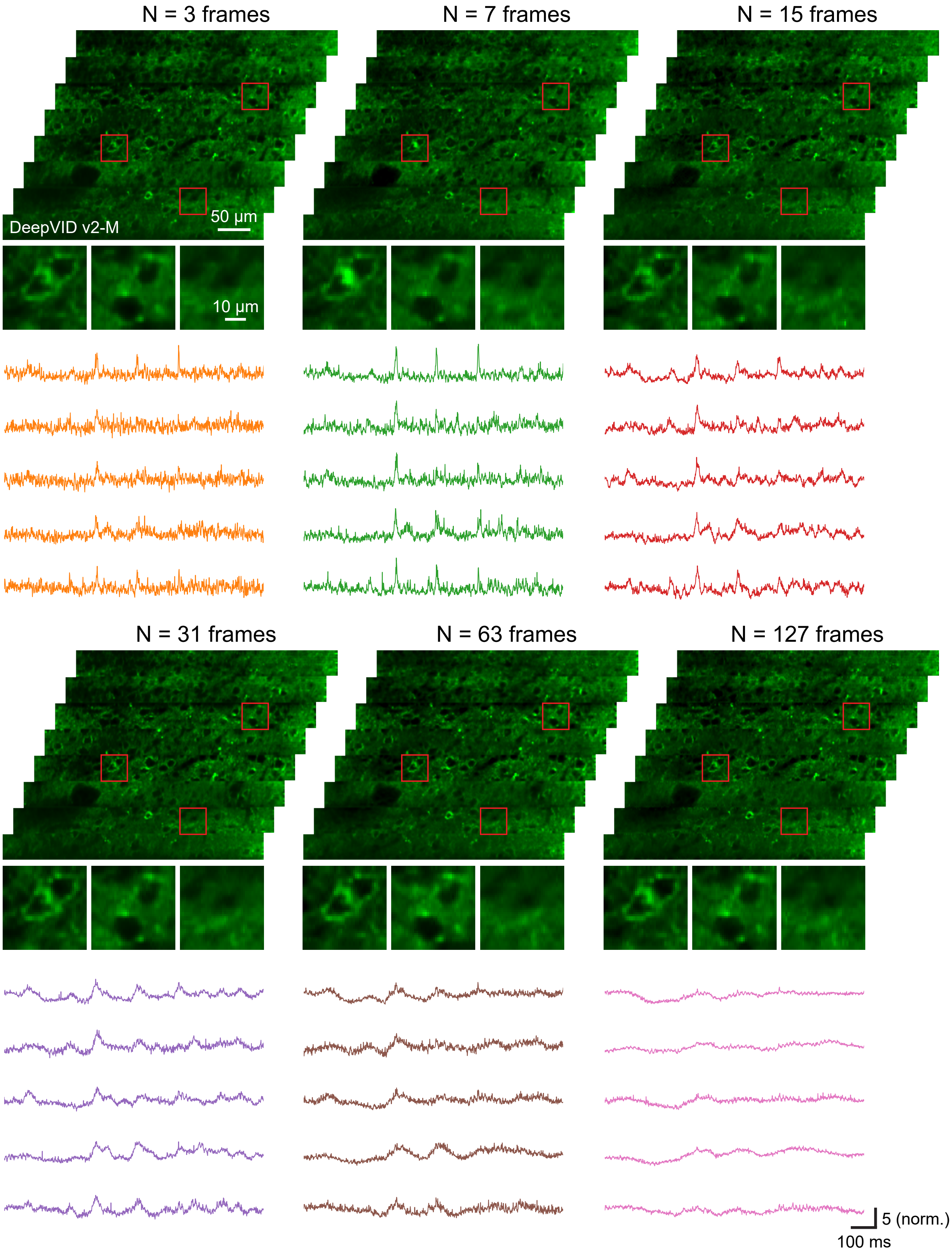

Supplementary Fig 9 Qualitative visualization of parameter analysis. Single-frame images and ROI time traces from DeepVID v2-M denoised videos trained with various input frames N from 3 to 127 frames, and all available frames for edge extraction (M).

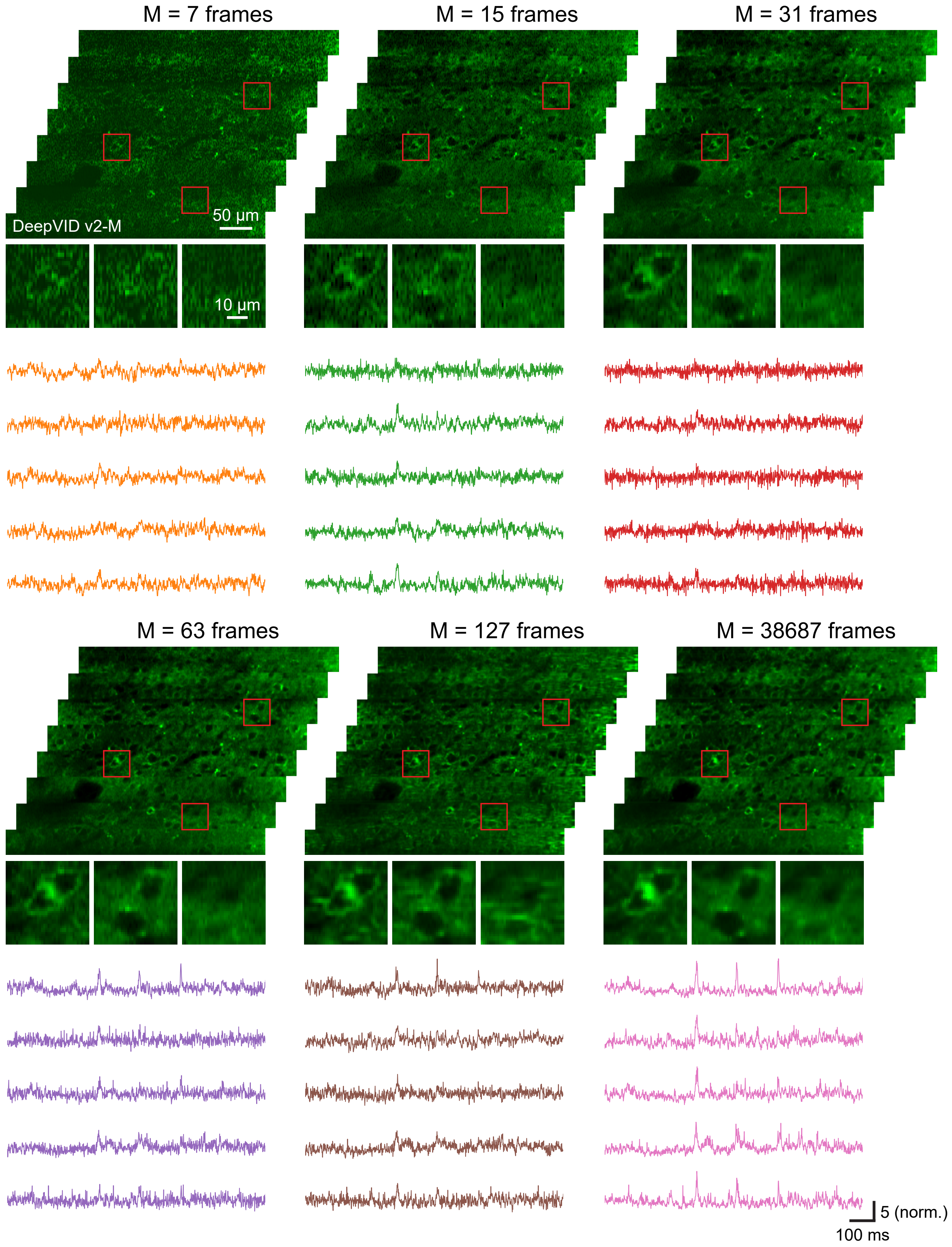

Supplementary Fig 10 Qualitative visualization of parameter analysis. Single-frame images and ROI time traces from DeepVID v2-M denoised videos trained with fixed input frames N at 7 frames, and various frames for edge extraction (M) from 7 to the maximum available frames.

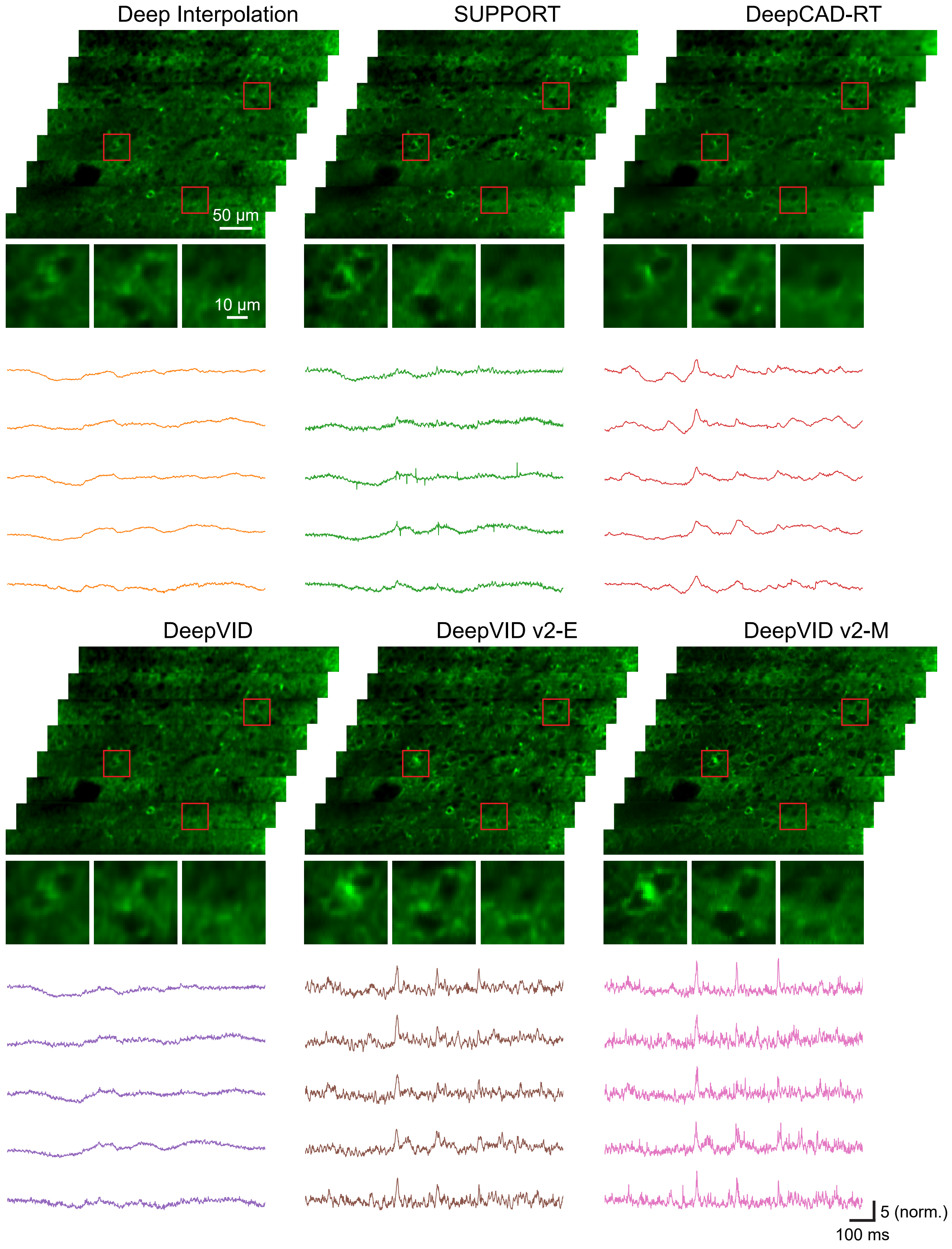

Supplementary Fig 11 Qualitative visualization of benchmark comparison. Single-frame images and ROI time traces from the raw and denoised videos. All benchmarks except DeepVID v2-E and DeepVID v2-M utilize $N = 127$ frames as input to the network. Both DeepVID v2-E and DeepVID v2-M maintain the optimal parameter settings with $N = 7$ and M using all available frames, due to the advantage of two adjustable parameters.

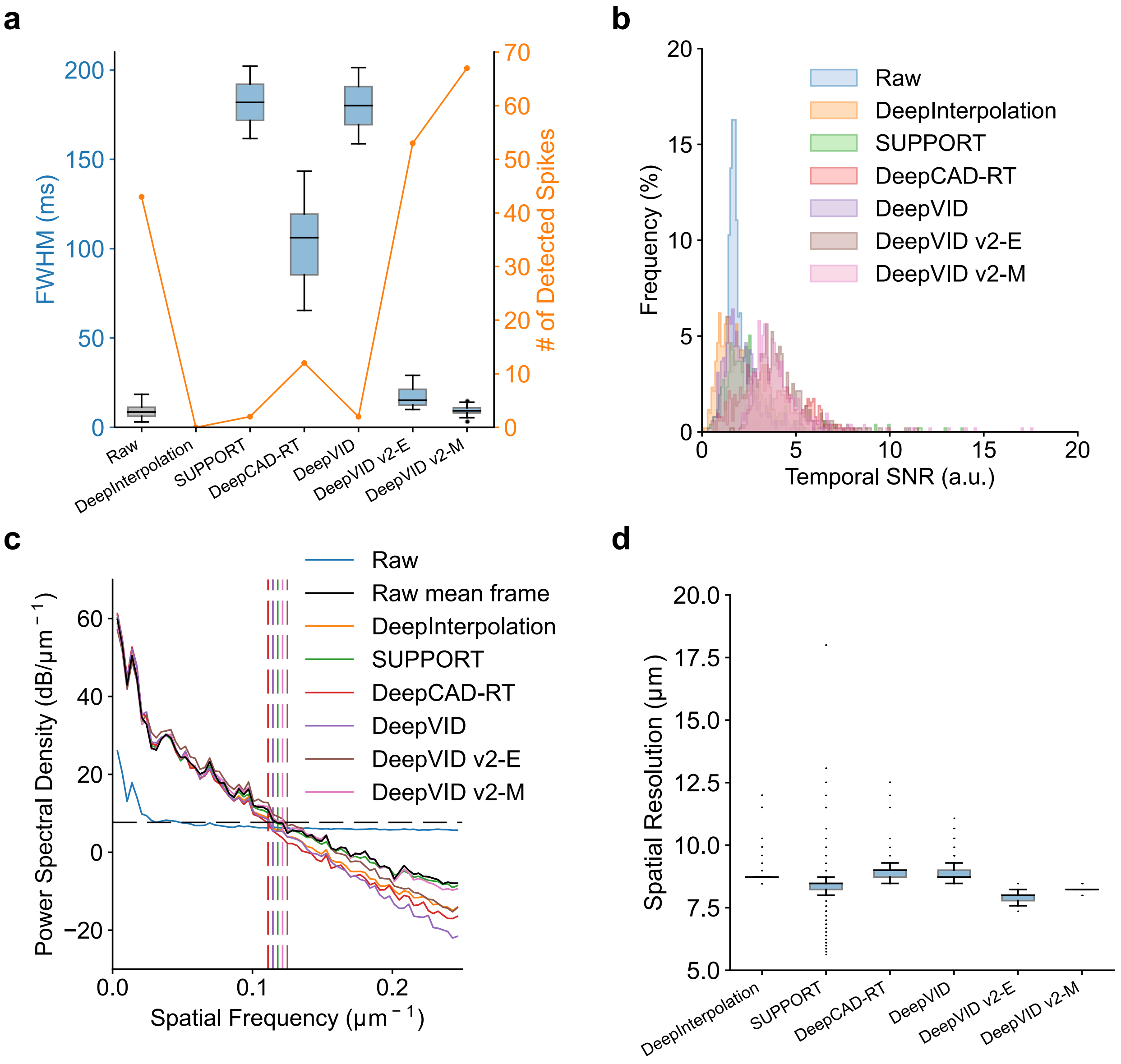

Supplementary Fig 12 Quantitative evaluation of benchmark comparison. (a) The number of detected spikes and FWHM of the detected spikes from the raw and denoised ROI time traces. (b) The histogram of temporal SNR of raw and denoised ROI time traces. (c) Power spectral density from single frames in raw and denoised videos, averaged over frames. (d) Spatial resolution of single frames in raw and denoised videos. All benchmarks except DeepVID v2-E and DeepVID v2-M utilize $N = 127$ frames as input to the network. Both DeepVID v2-E and DeepVID v2-M maintain the optimal parameter settings with $N = 7$ and M using all available frames, due to the advantage of two adjustable parameters.

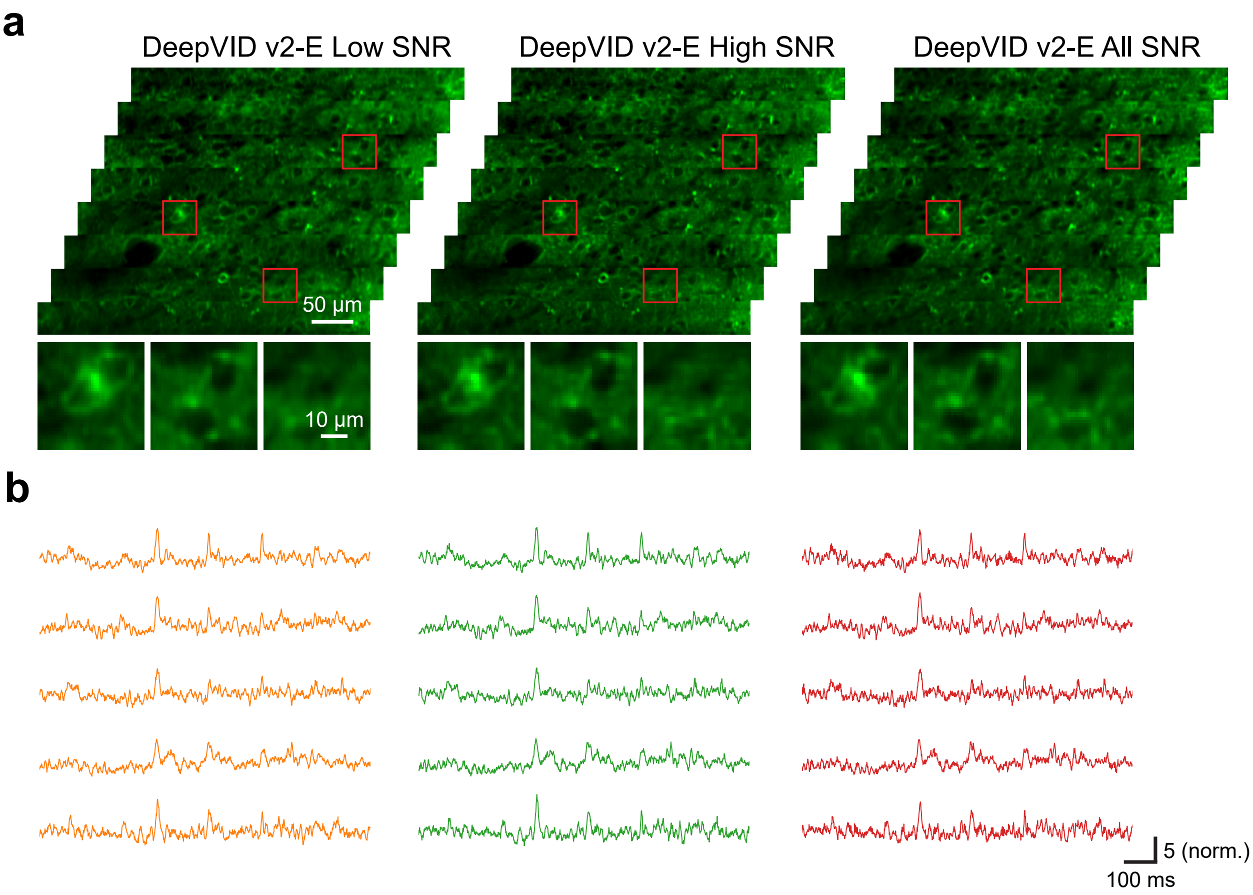

Supplementary Fig 13 Qualitative visualization of generalization. Single-frame images and ROI time traces from DeepVID v2-E denoised videos trained with each subset divided by temporal SNR and with the entire dataset.

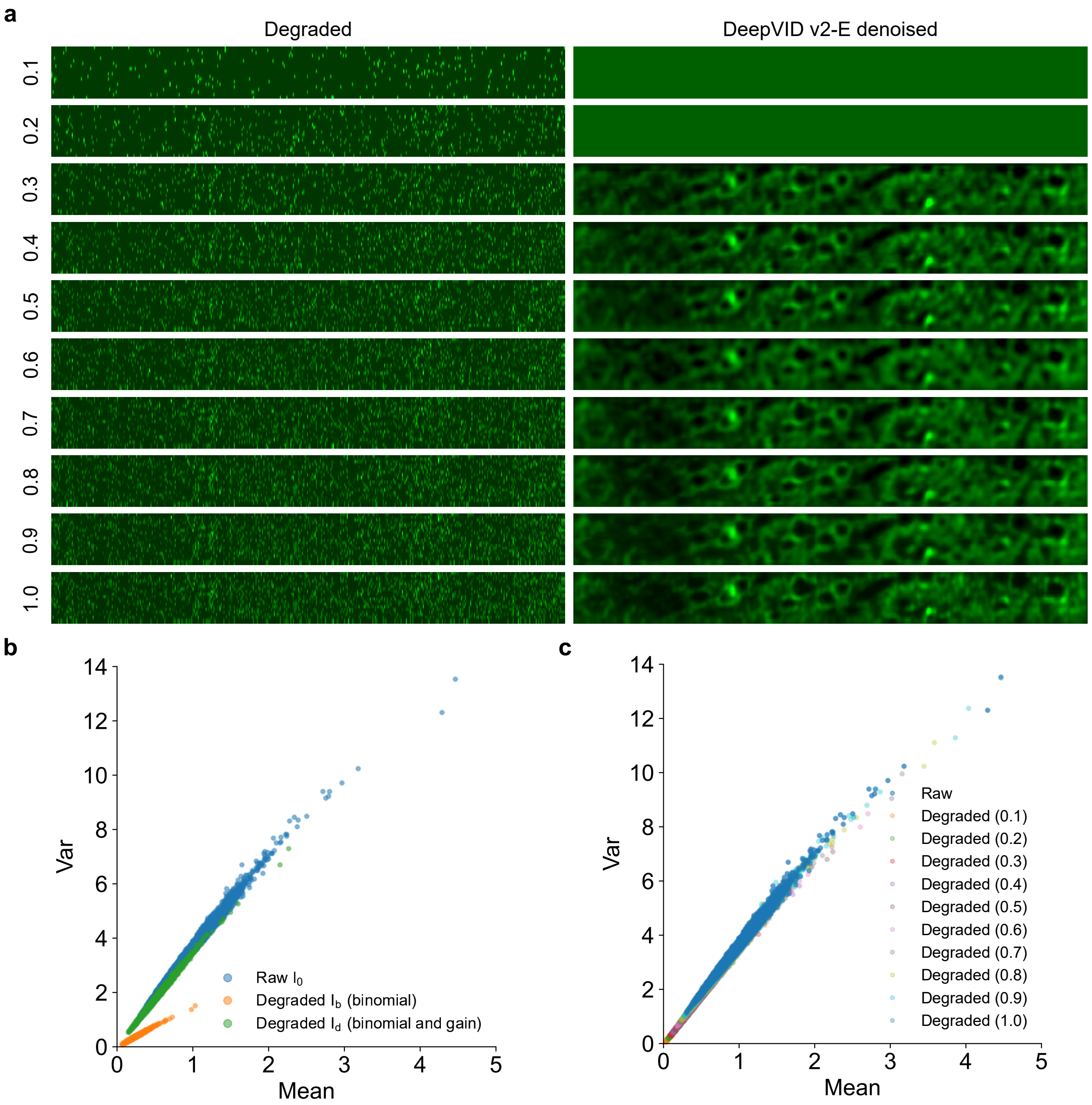

Supplementary Fig 14 Simulation of DeepVID v2 denoising capability in extreme low-photon regimes. (a) Single-frame images from degraded and DeepVID v2-E denoised videos at various photon levels. (b) Characteristic of noise in the raw and degraded videos after each step. (c) Characteristic of noise in the raw and degraded videos at various photon levels.

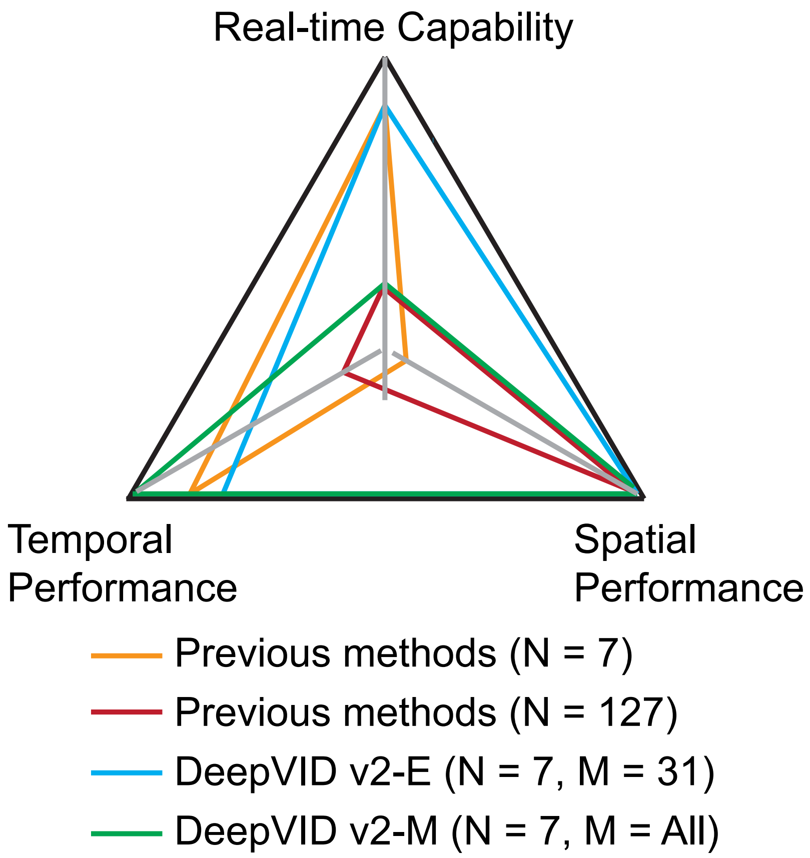

Supplementary Fig 15 Comparison of temporal performance, spatial performance and real-time capability for DeepVID v2-E, DeepVID v2-M, and previous methods.
